# Attitudes of US medical trainees towards neurology education: "Neurophobia" - a global issue

**DOI:** 10.1186/1472-6920-10-49

**Published:** 2010-06-23

**Authors:** Andrey V Zinchuk, Eoin P Flanagan, Niall J Tubridy, Wendy A Miller, Louise D McCullough

**Affiliations:** 1 Department of Medicine, Beth Israel Deaconess Medical Center, 330 Brookline Avenue, Boston MA, 02215 USA; 2 Department of Neurology, Mayo Clinic, 200 First St. S.W., Rochester MN 55905 USA; 3 Department of Neurology, St. Vincent's University Hospital, Elm Park 4. 01 2695005 Dublin, Ireland; 4 Department of Medicine, University of Connecticut Health Center, 263 Farmington Avenue, Farmington CT, 06030 USA; 5 Department of Neurology, University of Connecticut Health Center 263 Farmington Avenue, Farmington CT, 06030 USA; 6 The Stroke Center at Hartford Hospital, Hartford, CT, USA

## Abstract

**Background:**

Several studies in the United Kingdom and Asia have suggested that medical students and residents have particular difficulty in diagnosing and managing patients with neurological problems. Little recent information is available for US trainees. We examined whether students and residents at a US university have difficulty in dealing with patients with neurological problems, identified the perceived sources of these difficulties and provide suggestions for the development of an effective educational experience in neurology.

**Methods:**

A questionnaire was administered to third and fourth year medical students at a US school of medicine and to residents of an internal medicine residency program affiliated with that school. Perceived difficulties with eight medical specialties, including neurology, were examined. Methods considered to be most useful for learning medicine were documented. Reasons why neurology is perceived as difficult and ways to improve neurological teaching were assessed.

**Results:**

152 surveys were completed. Participation rates varied, with medical students having higher response rates (> 50%) than medical residents (27%-48%). Respondents felt that neurology was the medical specialty they had least knowledge in (p < 0.001) and was most difficult (p < 0.001). Trainees also felt they had the least confidence when dealing with patients with neurological complaints (p < 0.001). Residents felt more competent in neurology than students (p < 0.001). The paramount reasons for perceived difficulties with neurology were the complexity of neuroanatomy, limited patient exposure and insufficient teaching. Transition from pre-clinical to clinical medicine led to a doubling of "poor" ratings for neurological teaching. Over 80% of the respondents felt that neurology teaching could be improved through greater exposure to patients and more bedside tutorials.

**Conclusions:**

Medical students and residents at this US medical university found neurology difficult. Although this is consistent with prior reports from Europe and Asia, studies in other universities are needed to confirm generalizability of these findings. The optimal opportunity for improvement is during the transition from preclinical to clinical years. Enhanced integration of basic neurosciences and clinical neurology with emphasis on increased bedside tutorials and patient exposure should improve teaching. Studies are needed to quantify the effect of these interventions on confidence of trainees when dealing with patients presenting with neurological complaints.

## Background

Disorders of the nervous system are responsible for a remarkable 28% of all years of life lived with a disability [1]. As our population ages, the prevalence and public health impact of neurological diseases will continue to rise [2,3]. An increasing number of patients with neurological diseases are managed by hospitalists and primary care physicians (PCPs). In fact, the majority of these patients are managed by PCPs in the community and are never referred to a neurologist [3-5]. Thus, it is critically important that current and future hospital and community based physicians are comfortable with and competent in the basic management of patients with neurological illnesses. Equally important is that hospitalists and PCPs recognize when a patient requires referral for specialized care.

Several studies suggest that medical students and residents alike have particular difficulty in identifying and managing patients with neurological problems [6-8]. Recent data from the United Kingdom and Ireland show that students and junior physicians find neurology to be the most difficult among the medical specialties studied [3,4]. They feel they have a limited knowledge in this field, and express lack of confidence in their ability to deal with patients with neurological complaints [3,6]. Explanations for these findings varied from sense of intimidation by the perceived complexity of neurosciences to the limited exposure and poor teaching many trainees experience during the preclinical and clinical years [3,6]. These studies have prompted efforts to change curricula and clinical training in the UK and Ireland to better prepare students and residents for subsequent clinical practice [1,6,9].

No current data exists on the perceived competence and knowledge of neurology amongst United States (US) medical students and residents, as the one publication that identified problems with neurological education in the US dates back to 1984 [8]. Attitudes of current US trainees with regard to their knowledge of and clinical competence in neurology is critical information for those involved in medical education. By 2020 the demand for neurologists in the US will outstrip the supply by 20% and more patients will rely on their PCPs and hospitalists for their neurological care [10]. Future physicians will need to have a certain level of comfort in managing these patients.

In this study we evaluated the attitudes of medical students and residents toward neurology and neurological education at one US medical school and internal medicine residency program. The goals were to (i) determine whether students and residents perceive that they have difficulty in diagnosing and managing patients with neurological diseases (ii) identify the sources of these difficulties and (iii) provide data that could help design curricula for more effective education in neurology.

## Methods

This was a single center study that examined attitudes towards neurological education and perceived competency in dealing with patients with neurological complaints among medical trainees. Those surveyed included medical students in their clinical years and residents enrolled in an internal medicine program. A brief questionnaire (included as additional file 1), modeled after a survey used in an analogous study was utilized [4]. This study was approved by the IRB at The University of Connecticut Health Center.

The questionnaire contained three parts. Part 1 assessed the participants' perceived level of *knowledge, degree of difficulty *and *confidence in assessing and treating patients *in eight specialties commonly encountered in hospital and primary care settings. The specialties included were cardiology, gastroenterology, pulmonary, neurology, rheumatology, endocrinology, geriatrics and nephrology. A Likert scale was used to grade participants' responses. For example, in assessing participant's level of *knowledge *in each area of medicine a scale of 1 (Very Limited) to 5 (Very Good) was used.

In Part 2, participants were asked to rate the how useful they find various *methods for learning medicine*. A Likert scale and categorical items were used.

Part 3 of the survey evaluated participants' perceptions of neurology and neurological education. We assessed respondents' *exposure to neurological patients (number seen per year)*. Participants were then asked to rate several reasons *why neurology is difficult *for them (based on responses from previous studies [4]) and rate the *teaching of neurology *in pre-clinical, clinical and postgraduate settings. Finally, participants rated *how education in neurology can be improved*. Likert scale items as well as "open ended" questions were used.

Third and fourth year medical students and residents in the Internal Medicine program at a state university were surveyed between March and April of 2008. Participants were recruited via email broadcast messages and advertisements at clinical rotation locations. Survey responses were anonymous and all participants were provided with a statement regarding the voluntary and confidential nature of participation in the study. The project was approved by an Institutional Review Board.

The data were coded and analyzed using Statistical Package for Social Sciences (SPSS Inc. Chicago, IL, USA). General linear model multivariate analysis was used to compare the means of overall (student and resident) responses. A standard alpha level of 0.05 for significance threshold was used. A Bonferroni correction for multiple comparisons (alpha divided by n, where n is the number of comparisons being made) was used and the significance threshold for each comparison is presented along with the results. To compare the means of responses between medical students and residents, independent t-test was used with Bonferroni correction for multiple comparisons. For associations between categorical variables, Chi-square analysis was used.

## Results

A total of 152 surveys were completed. Participation from each subgroup was as follows: third year medical students 52 of 98 (53%), fourth year medical students 46 of 82 (56%), first year residents 24 of 50 (48%), second year residents 15 of 56 (27%) and third year residents 15 of 56 (27%). Respondents rated their knowledge of neurology as the lowest of the eight medical specialties (Table 1, p < 0.001). Neurology was also rated as the most difficult discipline (Table 1, p < 0.001) with the exception of nephrology, for which the difficulty rating was not statistically different from neurology (p = 0.141). Perhaps most revealing was that participants had the least confidence when assessing, diagnosing and treating patients presenting with neurological problems (p < 0.001) compared to other medical specialties.

**Table 1:** Ratings of knowledge, difficulty, and confidence for eight medical specialties

Specialty/Category	Knowledge		Difficulty		Confidence	
	Mean (SE)	P value	Mean (SE)	P value	Mean (SE)	P value
Cardiology	3.5 (0.1)	< 0.001	2.6 (0.1)	< 0.001	3.3 (0.1)	< 0.001
Gastroenterology	3.5 (0.1)	< 0.001	3.2 (0.1)	< 0.001	3.4 (0.1)	< 0.001
Respiratory	3.5 (0.1)	< 0.001	2.9 (0.1)	< 0.001	3.4 (0.1)	< 0.001
Neurology	2.7 (0.1)	n/a	2.3 (0.1)	n/a	2.6 (0.1)	n/a
Rheumatology	3.1 (0.1)	< 0.001	3.0 (0.1)	< 0.001	2.9 (0.1)	< 0.001
Endocrinology	3.4 (0.1)	< 0.001	3.0 (0.1)	< 0.001	3.3 (0.1)	< 0.001
Geriatrics	3.3 (0.1)	< 0.001	3.5 (0.1)	< 0.001	3.5 (0.1)	< 0.001
Nephrology	3.3 (0.1)	< 0.001	2.4 (0.1)	0.141	2.9 (0.1)	< 0.001

**Knowledge ratings: **1 - very limited, 2 - limited, 3 - moderate, 4 - good, 5 - very good.

**Difficulty ratings: **1 - very difficult, 2 - difficult, 3 - moderate, 4 - easy, 5 - very easy.

**Confidence ratings: **1 - very uncertain, 2 - uncertain, 3 - moderately confident, 4 - confident, 5 - very confident. P values from a generalized linear model comparing neurology to other specialties; significance threshold adjusted for multiple comparisons via Bonferroni correction (p < 0.007 denotes significance). n/a - not applicable. SE - standard error.

The perceived knowledge of neurology improved as clinical training advanced. Residents ranked their knowledge in neurology as higher than the medical students (3.0 vs. 2.5 respectively, p < 0.001). There were no differences in knowledge between the two groups for any of other specialties. The residents also perceived neurology to be less difficult than students (2.7 vs. 2.1 respectively, p < 0.001) and felt significantly more confident in dealing with patients with neurological problems (3.0 vs. 2.3 respectively, p < 0.001). Similar findings were true for nephrology (2.8 vs. 2.2 and 3.2 vs. 2.8 for difficulty and confidence respectively, p = 0.001). The students' and residents' confidence was equivalent for all other specialties (p = 0.04 - 0.899, p = 0.007 denotes threshold for significance).

In the second section of the questionnaire, we asked participants to rate how they learn medicine. Most trainees (73-75%) reported that online resources, textbooks and bedside teaching were useful or extremely useful, while only 57% and 46% placed learning from lectures and peers in either of these categories. When comparing each teaching method, learning through lectures was significantly less useful than the other top three methods (p < 0.012) while learning from peers was reported to be even less effective than that of lecture based teaching (p = 0.001, p = 0.013 denotes threshold for significance).

There were no significant differences in attitudes towards educational methods as responders advanced in training (all p values > 0.141), with the exception of learning from peers which residents rated to be more useful compared to medical students (p = 0.004, p = 0.010 denotes threshold for significance).

The third section of the survey evaluated participants' exposure to patients with neurological complaints, perceptions of neurology and neurological education in particular.

On a per year basis, 72% of participants reported seeing 30 patients or less with neurological complaints, while a remarkable 24% reported seeing 10 or less patients per year. Medical students were exposed to significantly less patients with neurological complaints compared to residents (X^2^, p = 0.01).

**Figure 1:**
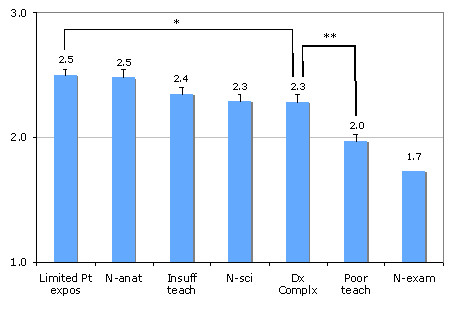
**Reasons contributing to why trainees find neurology difficult**. (Mean values, error bars indicate standard error; 1 = not at all, 2 = a minor contributor, 3 = a major contributor). * p value for comparison = 0.01, ** p value for comparison < 0.001, p = 0.008 denotes threshold for significance). N-atan - neuroanatomy; N-sci - neuroscience; Dx Complx - diagnostic complexity; Limited Pt expos - limited patient exposure; Insuff teach - insufficient teaching; Poor teach - poor teaching; N-exam - neurological examination.

Figure 1 illustrates the reasons participants found neurology to be difficult. These reasons were similar among medical students and residents except for the contribution of neuroanatomy, which residents found to be less of a contributor (2.2 [0.1] vs. 2.6 [0.1] for residents and medical students respectively, p < 0.001). Respondents suggested 10 additional reasons for why they find neurology difficult. Fourteen of 24 responses were related to poor integration of neuroscience and clinical neurology, poor teaching during first two years of medical school or the lack of a required clerkship. Only 3 of the 152 participants said they did not find neurology difficult.

The overall trends of neurological teaching as trainees progress in professional development are shown in Table 2. The differences between means (data not shown) for teaching during various stages of development were significant (Post graduate versus pre clinical and clinical: p = 0.013 and 0.009 respectively, p = 0.025 denotes significance threshold).

**Table 2:** Overall rating of teaching of neurology during the various training settings

Training setting/Rating	Very Poor or Poor	Moderate	Very good or good
Preclinical	17%	572%	26%
Clinical	37%	42%	21%
Postgraduate	4%	54%	22%

Medical students ranked teaching consistently lower in comparison to residents (Table 3). The differences in opinions between students and residents were significant across training settings (p values < 0.004, p = 0.025 denotes significance threshold). Again, the quality of neurological education declined from preclinical to clinical years (p < 0.001), for both residents and students.

**Table 3:** Comparison of student and resident views of neurological teaching at various training settings

Training Level	Preclinical	Clinical	Postgraduate
MS	3.0 (0.1)	2.6 (0.1)	NA
Resident	3.4 (0.1)	3.2 (0.1)	3.0

MS - medical student; Mean ratings (standard error).

**Figure 2:**
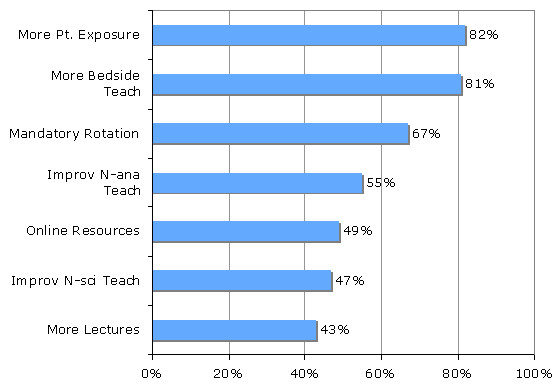
**Percentage of respondents rating each method to improve teaching as helpful or very helpful**. More Pt. Exposure = more patient exposure, More Bedside Teach - more bedside teaching, Improv N-ana Teach - improved neuroanatomy teaching, Online Resources - availability of online resources, Improv N-sci Teach - improved neuroscience teaching.

Participants reported that more teaching and more patient exposure were the most helpful methods to improve neurological teaching (Figure 2). A mandatory neurology rotation was also reported to be helpful, however significantly less so than increasing teaching and patient exposure (p = 0.002 and < 0.001 respectively). The remainder of methods for improvement of neurological teaching were rated as significantly less helpful than a mandatory rotation (p = 0.001 - 0.003, p = 0.008 denotes significance).

Participants felt that more lectures would be moderately helpful to helpful, although residents felt more strongly about the utility of lectures than students (p = 0.001). Improved neuroanatomy teaching was perceived to be significantly more helpful to residents than students (p = 0.006), as were neuroscience teaching (p = 0.001) and online resource use (p = 0.001, p = 0.007 denotes significance threshold).

## Discussion

This study represents the first structured investigation of neurological education in the United States in over 20 years. Since that time different methods for teaching neurology have been suggested [3,9,11]. The results of this study provide areas in which our teaching efforts can be improved and may help us design more effective curricula for neurological education.

Neurology is felt to be a difficult subject [1,4,6]. In our study, both medical students and residents perceived neurology as the most difficult medical specialty and the one they had least knowledge in (Table 1). Most importantly, participants felt least confident when diagnosing, assessing and treating patients with neurological disorders in comparison to seven other specialties commonly encountered in primary care settings (Table 1). These findings appear to be true across many learning systems. Investigators in Europe [4,6] obtained similar results and there are reports of "neurophobia" from Australia and Singapore as well [11,12]. These findings are important because this fear and lack of confidence may translate into practice. Although there is no data linking perception and performance directly, there is data suggesting that patients with common neurological illnesses such as seizures and back pain may be subject to suboptimal management and referral practices in primary care settings [9,13-15].

What are the reasons behind this discomfort with neurology? At what point during professional development does it occur? How can we improve the current situation?

Echoing previous reports [4,6], in our study, neuroanatomy was reported as one of the top reasons for why neurology is a difficult subject, especially amongst students. Over 50% of respondents stated that improvements in this area would be a helpful way to improve neurological teaching. Notably, neuroanatomy was a smaller contributor to the difficulty of neurology for residents compared to students. Assuming that for an average trainee, neuroanatomy knowledge is higher during medical school years than in residency training, these findings suggest that a detailed knowledge of neuroanatomy may not be essential for the practical management of patients with basic neurological problems. Indeed several authors have supported this idea and utilize the metaphor that "most people learn to drive safely with limited knowledge of how the engine works" [4,9]. Complexity of neurological diagnoses and basic neuroscience was also rated among the top five contributors to difficulty of neurology, in agreement with findings from the UK and Ireland [4,6]. Although detailed knowledge of the vast array of unusual neurological diagnoses and neuroscience underpinning them may be critical for seasoned, practicing neurologist, for a medical student, focus on the core neurology knowledge and skills may elicit more interest [6,9], and perhaps decrease anxiety about the subject. Based on our findings, a more clinically oriented approach to neuroanatomy and neuroscience teaching will be of greatest use.

The remaining two of the primary five reasons trainees found neurology difficult were 1) limited exposure to patients with neurological problems and 2) insufficient teaching (Figure 1). In fact, over 70% of participants reported seeing less than thirty patients with neurological complaints per year, with residents seeing significantly more than students. The positive impact of clinical exposure to patients with neurological complaints is highlighted by the fact that residents felt they had a higher degree of knowledge, less difficulty and more confidence with neurology than students (Table 1). It is unlikely that these improvements are simply due to general increase in comfort of managing all patients as one advances from being a medical student to resident, as no differences between student and resident ratings were found for any other specialty with the exception of nephrology (difficulty rating only).

Based on results in this study, it is not only the lack of exposure and teaching, but also the absence of appropriate integration of basic neurosciences and clinical neurology that poses an obstacle for trainees. The overall ranking of neurological teaching was moderate (Table 2), however during the transition from preclinical to clinical years, a doubling of "very poor or poor" teaching ratings occurred. This concerning trend was the same for both students and residents (Table 3) suggesting that the transition from preclinical to clinical years is the critical period for gaining functional knowledge of neurology. These findings provide additional evidence that it is imperative that educators improve integration of basic neuroscience, anatomy and clinical neurology [4,6,7,12].

Over 70% of trainees reported that bedside teaching, textbooks and online resources, unlike lectures, were very useful or extremely useful ways to learn medicine. This was true for both students and residents in our study. These findings suggest that across the educational developmental stages, an active learning process integrating acquisition of information, placement in context, and its practical use in patient encounters works best for learners. This is consistent with investigations of the amount of knowledge gained in relation to learning styles and clinical exposure among medical students [16]. Indeed, over 80% of trainees felt that more bedside teaching and increased patient exposure would be a "helpful to very helpful method" to improve neurological teaching. This strongly suggests that the number of small group tutorials and amount of clinical teaching should be increased [4,9].

The over arching findings of this study were a perception of insufficient clinical exposure and poor integration of preclinical and clinical neurological teaching amongst trainees. One way to achieve increased clinical exposure may be to institute a mandatory experience during medical school, including exposure to both inpatient and outpatient neurology. A number of different teaching methods have been suggested to improve competency in neurology [3,7]. It appears that the best results can be obtained by early integration of neuroanatomy, neuroscience and clinical neurology. Recent reports on case based teaching and caring for a "virtual neurological patient" showed that integration of clinical neurology and the neuroscience during the first two years of undergraduate medical education and early on in clerkship years can improve teaching and reduce "neurophobia" [11,12].

Also of note is the finding of how useful a vast majority of participants found online resources to be (Figure 2). Such results are consistent with prior studies in the US [17,18], but differ from findings in an Irish study [4], where only a small minority felt they learned most online. This disparity may reflect the better availability and thus greater use of online resources in the US versus Ireland, and highlights the differing challenges across the educational systems. Greater access to online resources may help improve neurological teaching in places like Ireland. In systems like the one in the U.S., incorporation of video tutorials, which has been shown to be an effective teaching method [19] as part of the online educational arsenal, may provide an economical supplement to increasing patient exposure and bedside teaching.

It should be noted that there are several limitations to this study. The response rates were relatively low, as is often seen in survey studies, which may increase bias. This was particularly notable as residents advanced in their training. In addition, the definitions of several terms within the survey such as "lectures" or "neurological complaint" may vary between students and residents, thus potentially creating bias in the ratings of their utility and exposure respectively. Another important limitation is related to the fact that this study was performed at a single medical institution, thus our findings may be difficult to generalize to other medical schools in the US, where variability in curricula and teaching faculty may lead to different results. It is notable however, that similar trends were found in Europe and Asia, suggesting that issues identified in this work may be true across educational establishments [4,6,11,12]. Nonetheless, studies at additional US medical centers are needed to confirm that the data presented here can be generalized to other medical training programs in the US. Perhaps most importantly this study only evaluated the perception of trainees' confidence with neurology. Studies are needed to determine if the perceived difficulty with neurology correlates with poor performance on written and patient care assessments. Investigations are also needed to assess if interventions such as greater patient exposure, increased clinical neurology teaching and improved integration of neuroscience and clinical neurology lead to a better performance on written or clinical evaluations.

## Conclusions

Both students and residents at the institution studied report significant difficulty and low confidence when working with patients with neurological complaints. This is especially concerning in the face of the rising number of patients with neurological disorders that are managed in many primary care settings. Although it is difficult to generalize these findings based on a single center study to the remainder of the US medical educational institutions, our findings are consistent with reports from diverse educational settings in Europe and Asia. Our data also suggests that trainee's perceptions are primarily due to lack of patient exposure, insufficient teaching and poor integration of clinical neurology and the neuroscience that forms its foundation. The needs in these areas should be addressed and the effectiveness of these interventions should be evaluated in future studies. This will serve as an important step forward towards training clinicians capable of meeting the rising challenges of managing patients with neurological disorders.

## Competing interests

The authors declare that they have no competing interests.

## Authors' contributions

AVZ, NJT, EPF and LDM conceived of the study. AVZ and WAM gathered the data. AVZ was responsible for statistical analysis and manuscript preparation. All authors participated in data interpretation, manuscript revision and final approval of version published.

## Pre-publication history

The pre-publication history for this paper can be accessed here:

http://www.biomedcentral.com/1472-6920/10/49/prepub

## Supplementary Material

Additional File 1 **Questionnaire utilized in this study**. Click here for file
