# Supplementary material for: Attitudes of US medical trainees towards neurology education: "Neurophobia" - a global issue
Source: BMC Med Educ. 2010 Jun 23;10:49. doi: 10.1186/1472-6920-10-49 (PMC2900283; doi:10.1186/1472-6920-10-49)
Supplement: Additional File 1 — Questionnaire utilized in this study. [file 1472-6920-10-49-S1.DOC]

**MEDICAL TEACHING QUESTIONNAIRE**

YOUR PARTICIPATION IN THIS SURVEY IS VOLUNTARY & ANONYMOUS.  WHETHER YOU PARTICIPATE OR NOT, WILL NOT IN ANY WAY INFLUENCE YOUR GRADES/PERFORMANCE EVALUATIONS. YOU MAY SKIP ANY QUESTION FOR ANY REASON. COMPLETION & RETURN OF THIS SURVEY IMPLIES CONSENT.

We are attempting to determine areas of difficulty among medical students, hospital doctors and general practitioners in an effort to improve medical teaching.

***SECTION 1:* Please Circle choices in questions below**

1. **How would you describe your knowledge in the following areas of Medicine**

|  | **Very Limited** | **Limited** | **Moderate** | **Good** | **Very Good** |
| --- | --- | --- | --- | --- | --- |
| **Cardiology** | 1 | 2 | 3 | 4 | 5 |
| **Gastroenterology** | 1 | 2 | 3 | 4 | 5 |
| **Respiratory** | 1 | 2 | 3 | 4 | 5 |
| **Neurology** | 1 | 2 | 3 | 4 | 5 |
| **Rheumatology** | 1 | 2 | 3 | 4 | 5 |
| **Endocrinology** | 1 | 2 | 3 | 4 | 5 |
| **Geriatrics** | 1 | 2 | 3 | 4 | 5 |
| **Nephrology** | 1 | 2 | 3 | 4 | 5 |

1. **How would you rate each of the following subjects in degree of difficulty?**

|  | **Very Difficult** | **Difficult** | **Moderate** | **Easy** | **Very Easy** |
| --- | --- | --- | --- | --- | --- |
| **Cardiology** | 1 | 2 | 3 | 4 | 5 |
| **Gastroenterology** | 1 | 2 | 3 | 4 | 5 |
| **Respiratory** | 1 | 2 | 3 | 4 | 5 |
| **Neurology** | 1 | 2 | 3 | 4 | 5 |
| **Rheumatology** | 1 | 2 | 3 | 4 | 5 |
| **Endocrinology** | 1 | 2 | 3 | 4 | 5 |
| **Geriatrics** | 1 | 2 | 3 | 4 | 5 |
| **Nephrology** | 1 | 2 | 3 | 4 | 5 |

1. **How confident would you be in assessing, diagnosing and treating patients presenting with problems related to each of the following specialties?**

|  | **Very Uncertain** | **Uncertain** | **Moderately Confident** | **Confident** | **Very Confident** |
| --- | --- | --- | --- | --- | --- |
| **Cardiology** | 1 | 2 | 3 | 4 | 5 |
| **Gastroenterology** | 1 | 2 | 3 | 4 | 5 |
| **Respiratory** | 1 | 2 | 3 | 4 | 5 |
| **Neurology** | 1 | 2 | 3 | 4 | 5 |
| **Rheumatology** | 1 | 2 | 3 | 4 | 5 |
| **Endocrinology** | 1 | 2 | 3 | 4 | 5 |
| **Geriatrics** | 1 | 2 | 3 | 4 | 5 |
| **Nephrology** | 1 | 2 | 3 | 4 | 5 |

***SECTION 2.***

1. **How useful do you find each of the below methods in learning medicine?**

|  | **Not**  **Useful** | **Somewhat Useful** | **Very**  **Useful** | **Extremely Useful** |
| --- | --- | --- | --- | --- |
| **Online Resources**  **(**e.g. tutorials, databases, cases, NOT online textbooks**)** | 1 | 2 | 3 | 4 |
| **Text books (**including online textbooks**)** | 1 | 2 | 3 | 4 |
| **Lectures** | 1 | 2 | 3 | 4 |
| **Bedside Tutorials** | 1 | 2 | 3 | 4 |
| **Peers** | 1 | 2 | 3 | 4 |
| **Other**  (leave blank if not applicable) | 1 | 2 | 3 | 4 |

*(If you selected Other, please specify)___________________________________*

1. **How helpful have you found your knowledge of the following pre clinical subjects when applied to clinical years?**

|  | **Very Unhelpful** | **Unhelpful** | **Moderately Helpful** | **Helpful** | **Very Helpful** |
| --- | --- | --- | --- | --- | --- |
| **Anatomy** | 1 | 2 | 3 | 4 | 5 |
| **Biochemistry** | 1 | 2 | 3 | 4 | 5 |
| **Histology** | 1 | 2 | 3 | 4 | 5 |
| **Physiology** | 1 | 2 | 3 | 4 | 5 |
| **Pharmacology** | 1 | 2 | 3 | 4 | 5 |
| **Pathology** | 1 | 2 | 3 | 4 | 5 |
| **Microbiology** | 1 | 2 | 3 | 4 | 5 |
| **Pathophysiology** | 1 | 2 | 3 | 4 | 5 |

1. **Current position**

(Please select ‘X’ box accordingly)

| **Medical Student Year 3** |  |
| --- | --- |
| **Medical Student Year 4** |  |
| **Intern (PGY-1)** |  |
| **Resident (PGY-2)** |  |
| **Resident (PGY-3)** |  |

1. **How would you rate your exposure (encounters *per year*) to *patients with neurological complaints*?**

(Please circle most appropriate category of patients encountered *per year*).

| **Category:** | **0 patients** | **1 – 10 patients** | **11 – 30 patients** | **31 – 100 patients** | **> 100 patients** |
| --- | --- | --- | --- | --- | --- |

***SECTION 3***

1. **How would you rate the following as reasons for Neurology being a difficult subject?**

|  | **Not at all** | **A Minor Contributor** | **A Major Contributor** |
| --- | --- | --- | --- |
| **Neuroanatomy** | 1 | 2 | 3 |
| **Neuroscience** | 1 | 2 | 3 |
| **The Clinical Neurological Exam** | 1 | 2 | 3 |
| **Poor Teaching** | 1 | 2 | 3 |
| **Many Complex Diagnoses** | 1 | 2 | 3 |
| **Limited Exposure to Neurological Patients** | 1 | 2 | 3 |
| **Not Enough Teaching** | 1 | 2 | 3 |

1. **Please List any other reasons as to why you think Neurology is a difficult subject.**

**________________________________________________________________________________________________________________________________________________________________________________________________________________________________________________________________________________________________**

1. **How would you rate the teaching of Neurology in each of the following subsets?**

|  | **Very Poor** | **Poor** | **Moderate** | **Good** | **Very Good** |
| --- | --- | --- | --- | --- | --- |
| **Pre-Clinical** | 1 | 2 | 3 | 4 | 5 |
| **Clinical** | 1 | 2 | 3 | 4 | 5 |
| **Postgraduate**  (Leave blank if not applicable) | 1 | 2 | 3 | 4 | 5 |

1. **Please indicate how each of the following would be helpful in improving neurological teaching?**

|  | **Very Unhelpful** | **Unhelpful** | **Moderately Helpful** | **Helpful** | **Very Helpful** |
| --- | --- | --- | --- | --- | --- |
| **More bedside teaching** | 1 | 2 | 3 | 4 | 5 |
| **More patient exposure** | 1 | 2 | 3 | 4 | 5 |
| **More lectures** | 1 | 2 | 3 | 4 | 5 |
| **A *mandatory* Neurology rotation for medical students** | 1 | 2 | 3 | 4 | 5 |
| **Improved neuroanatomy teaching** | 1 | 2 | 3 | 4 | 5 |
| **Improved neuroscience teaching** | 1 | 2 | 3 | 4 | 5 |
| **Availability of online tutorials/resources** | 1 | 2 | 3 | 4 | 5 |
| **Other**  (Please specify below or leave blank if N/A) | 1 | 2 | 3 | 4 | 5 |

Other ____________________________________________________
